# Bioinformatics-Structural Approach to the Search for New D-Amino Acid Oxidases

**DOI:** 10.32607/actanaturae.11812

**Published:** 2022

**Authors:** D. L. Atroshenko, D. I. Golovina, E. P. Sergeev, M. D. Shelomov, A. G. Elcheninov, I. V. Kublanov, T. A. Chubar, A. A. Pometun, S. S. Savin, V. I. Tishkov

**Affiliations:** Department of Chemistry, Lomonosov Moscow State University, Moscow, 119991 Russia; Federal Research Centre “Fundamentals of Biotechnology” of RAS, Moscow, 119071 Russia

**Keywords:** D-amino acid oxidase, primary structure, ternary structure, modelling, AlphaFold 2, glycine oxidases

## Abstract

D-amino acid oxidase (DAAO, EC 1.2.1.2) plays an important role in the functioning of prokaryotes as well as of lower (yeast and fungi) and higher eukaryotes (mammals). DAAO genes have not yet been found in archaean genomes. D-amino acid oxidase is increasingly used in various fields, which requires the development of new variants of the enzyme with specific properties. However, even within one related group (bacteria, yeasts and fungi, mammals), DAAOs show very low homology between amino acid sequences. In particular, this fact is clearly observed in the case of DAAO from bacteria. The high variability in the primary structures of DAAO severely limits the search for new enzymes in known genomes. As a result, many (if not most) DAAO genes remain either unannotated or incorrectly annotated. We propose an approach that uses bioinformatic methods in combination with general 3D structure and active center structure analysis to confirm that the gene found encodes D-amino acid oxidase and to predict the possible type of its substrate specificity. Using a homology search, we obtained a set of candidate sequences, modelled the tertiary structure of the selected enzymes, and compared them with experimental and model structures of known DAAOs. The effectiveness of the proposed approach for discrimination of DAAOs and glycine oxidases is shown. Using this approach, new DAAO genes were found in the genomes of six strains of extremophilic bacteria, and for the first time in the world, one gene was identified in the genome of halophilic archaea. Preliminary experiments confirmed the predicted specificity of DAAO from Natronosporangium hydrolyticum ACPA39 with D-Leu and D-Phe.

## INTRODUCTION

Any cell is a highly complex open-type multienzyme system, and depending on the complexity and specific state of functioning of the organism, the same enzyme can perform different physiological roles. A good example is D-amino acid oxidase (DAAO, EC 1.4.3.3). In bacteria, yeast, and fungi, the main role of this enzyme is limited to the utilization of exogenous D-amino acids (primarily D-Ala) [1, 2]. In higher eukaryotes – vertebrates and especially in mammals, the main role of DAAO is to maintain a certain level of D-amino acids, which are regulators of many important processes, primarily nervous activity. For example, a decrease in the level of D-Ser in the cerebrospinal fluid due to increased DAAO activity is associated with schizophrenia [3, 4]. In Alzheimer’s and Parkinson’s diseases, increased levels of D-Ala are observed in nervous tissues [4, 5]. Therefore, the search for effective and specific inhibitors of human DAAO seems to be relevant. D-amino acid oxidase is also widely used in practice [6, 7, 8, 9]. For example, DAAO from the yeast Trigonopsis variabilis is used in the two-enzyme biocatalytic process for the production of 7-aminocephalosporonic acid (7-ACA) from cephalosporin C [10, 11]. This process reduces the consumption of organic solvents by 400 times compared to the previously used purely chemical method. The production of 7-ACA, used as a synthone for the production of semisynthetic cephalosporins of various generations, reaches several thousand tons per year.

The practical application of the enzyme requires the use of a biocatalyst with certain properties. There is no universal enzyme in nature. Its activity and specificity are stipulated by the role it per forms in nature. In most biotechnological processes, the substrates and reaction conditions differ from those in nature. Therefore, when developing a new process, method of analysis and in other cases for each one, the properties of the biocatalyst are adjusted to the requirements of the process. This is usually done by protein engineering methods. Obviously, the enzyme whose properties are the closest to the required ones seems to be the optimal starting object. For this purpose, genes are searched for in sequenced genomes, the number of which is constantly increasing. The genes of D-amino acid oxidases from yeast and fungi were cloned, and the properties of the enzymes were studied from only 7 sources: Fusarium solani (FsoDAAO) [12], Trigonopsis variabilis (TvaDAAO) [13], Rhodosporidium toruloides (formerly Rhodotorula gracilis) (RtoDAAO) [14], Pichia pastoris (PpaDAAO) [15], Candida boidinii (CboDAAO) [16], Rasamsonia emersonii strain YA (RemDAAO) [17], and Ogataea parapolymorpha DL-1 [18]. In the latter case, the genes of five different DAAOs (OpaDAAO1 – OpaDAAO5) and one D-aspartate oxidase (DASPO) were identified, cloned, and expressed in E. coli. In the case of bacteria, only three enzymes have been cloned and described thus far: from Rubrobacter xylanophilus (RxyDAAO), Streptomyces coelicolor (ScoDAAO), and Arthrobacter protophormiae (AprDAAO) [9]. Currently, there are no data on the presence of potential daao genes in the archaean genomes in the literature and databases. The main reason for this state of affairs in the research and application of bacterial DAAOs is the difficulty in finding enzyme genes in bacterial genomes. A total of just over 10 DAAO genes have been identified, and all of them have been found in the genomes of bacteria belonging to Acinetobacteria [7, 9]. The difficulty of the search is related to the fact that the amino acid sequences of DAAOs are highly variable. Therefore, the traditional widely used homology search is a very difficult task. In addition, there is a closely related enzyme, glycine oxidase (GOX), which very often appears when searching for DAAO by homology with known enzymes of this type.

The second important point in the search for new DAAOs is the selection of candidates with properties closest to those needed. Common DAAOs, with the exception of the highly specific D-aspartate oxidase, exhibit broad substrate specificity. Depending on the source, the spectrum of substrate specificity varies greatly, and activity with different D-amino acids may differ by an order of magnitude or more. Moreover, in some cases, the substrate specificity of DAAOs may have special requirements. For example, when developing methods for diagnosing neurodegenerative diseases, DAAOs that are active with D-Ser but not with D-Ala and vice versa are required [5]. Therefore, to select a DAAO with the desired substrate specificity (if its description is not available), we have to clone and express a representative set of enzymes, purify and study their catalytic properties and select the best one. Obviously, this procedure is laborious, time-consuming, and expensive.

We have proposed a bioinformatic-structural approach that allows us to show with high reliability the belonging of candidate enzymes exactly to DAAOs, discriminate them from glycine oxidases, and, using the correlation between substrate specificity and experimental or model structures of known DAAOs, make a reasonable assumption about the spectrum of substrate specificity. Particular emphasis is placed on using data on enzymes from the thermotolerant yeast O. parapolymorpha DL-1 (five OpaDAAO and OpaDASPO) because five of the six enzymes exhibit unusual dependencies of stability and activity on medium pH and have a very interesting and promising spectrum of substrate specificity. This approach has been successfully tested on a number of sequences from extremophilic bacteria. The presence of the daao gene in the genome of a halophilic archaea has been shown for the first time in the world.

## EXPERIMENTAL

**Bioinformatic search for potential DAAO genes**

The homology search for new DAAOs was performed using BLASTp software (https://blast.ncbi.nlm.nih.gov/ Blast.PAGE=Proteins) against a database of translated protein sequences from the genomes of extremophilic bacteria. The UniProt NCBI was used as the main source. We also searched the genomes of bacteria and archaea whose sequences were determined during work under Agreement No. 075-15-2021-1396 dated 10/26/2021 (Federal Research Program for the Development of Genetic Technologies for 2019–2027). The sequences that showed the highest homology were selected for further work.

Multiple alignment of the selected sequences and a number of known bacterial and yeast sequences was carried out with Clustal X 1.83 software.

**Construction and analysis of DAAO model structures**

The open-access online server for AlphaFold2 was used to build model structures of enzymes [19, 20]. MMseqs2 software was used for multiple alignments, and three cycles of prediction refinement were performed for each model. Five models were generated, and the best variant was chosen based on the pLDDT value [19]. All obtained structures had a pLDDT greater than 90. The FAD molecule was incorporated by optimizing the position in the globule and bond geometry with Coot software [21].

Substrate docking was performed using AutoDock software [22] with GPU acceleration [23]. The following parameters were used for docking: ga_pop_size = 150, ga_num_evals = 25000000, ga_run = 20, ga_mutation_rate 0.02–0.08, Solis-Wets method. Docking results were selected based on the positions of the carboxyl group, amino group, and Cα-atom of the D-amino acid suitable for catalysis of reaction. The corresponding positions were selected based on the crystal structures of RtoDAAO in complex with D-alanine/iminopyruvate (PDBID 1C0P) and pkDAAO (from pig kidney) in complex with iminotryptophan (PDBID 1DDO). The position of the D-amino acid side chain was chosen based on the potential interactions of the substrate with DAAO.

The RMSD between structures was calculated by Cα atoms using the “align” command of the PyMol software package (The PyMOL Molecular Graphics System, Version 2.1.0, Schrödinger, LLC). Five cycles of structural emission deviations (parameter “cycles”) were used to calculate the RMSD.

The structures were also visualized using PyMol software (The PyMOL Molecular Graphics System, Version 2.1.0, Schrödinger, LLC).

## RESULTS AND DISCUSSION

**Homology search for new DAAOs from extremophilic bacteria and archaea**

The search for new potential DAAOs was performed using the UniProt NCBI database for bacterial genomes and the joint database of sequenced genomes of extremophilic microorganisms of Lomonosov Moscow State University and Federal Research Centre “Fundamentals of Biotechnology” of the Russian Academy of Sciences (FRC Biotechnology RAS). Amino acid sequences of enzymes from the yeast R. toruloides (better known as R. gracilis), T. variabilis, C. boidinii, O. parapolymorpha DL-1 (five DAAOs and one DASPO) and the bacteria A. protophormiae, R. xylanophilus and S. coelicolor were used as references. Detailed information about the sources of DAAO sequences used in this work is presented in Table 1. In the case of bacteria, sequences of only those enzymes with proven oxidase activity were used. First, the new enzymes were compared with the most well-studied DAOOs from R. toruloides and T. variabilis. Special attention was given to five DAAOs and one DASPO from the yeast O. parapolymorpha DL-1 because this is the only organism thus far in which so many paralogous enzymes have been obtained and studied. The daao and daspo genes from the yeast O. parapolymorpha DL-1 were cloned and expressed in E. coli cells in the active form. Four DAAOs and DASPO were obtained in a highly purified form, their catalytic parameters with D-amino acids were determined, their activity and stability dependencies were studied at different medium pH values, and their thermal stability at pH values optimal for stability was studied. The amino acid sequences of vertebrate DAAO were not used because they initially had low homology with microbial enzymes [1, 2, 9].

The search for DAAO homologues in bacterial genomes deposited at UniProt NCBI found a large number of candidate sequences, but the level of homology did not exceed 30%. An expert evaluation of the search results showed that the vast majority of sequences with a homology level of less than 23% cannot be attributed to DAAO. Therefore, only sequences from thermophilic bacteria with homology levels of 24–30% were selected for further work. Expert evaluation of these proteins based on conserved residues (see the next section) allowed us to narrow down the set to the sequences characteristic of DAAO and GOX. A similar sequence of procedures was used when searching for potential DAAO genes in the genomes of extremophiles and archaea in the database of Lomonosov Moscow State University and the FRC Biotechnology RAS.

**Comparison of the amino acid sequences of the new DAAOs with known enzymes from bacteria, yeast, and fungi**

**Fig. 1:**
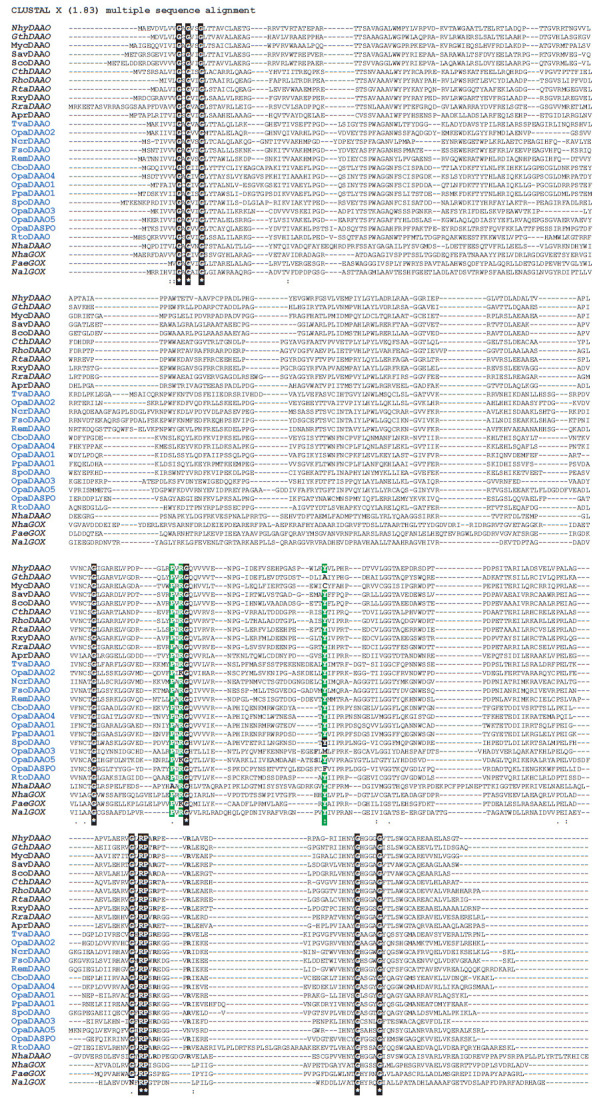
Alignment of the amino acid sequences of D-amino acid oxidases from yeast, bacteria and archaea (the names are shown in blue and black, respectively). See *Table 1* for the name correspondence. Novel DAAO sequences from bacteria analysed in this work are shown in bold italics. Letters on a green background in the alignment refer to residues that were previously considered as conserved for D-amino acid binding in the DAAO active site

Figure 1 shows some of the results of multiple alignment of the identified sequences (names of new enzymes are in bold italics) with the sequences of reference DAAOs. To avoid cluttering the already large figure, it does not show all the search results in the UniProt NCBI database. We left only the sequences of five DAAOs from thermomophilic microorganisms and did not provide data for glycine oxidases. However, multiple alignments of the glycine oxidase sequences were also performed, and model structures were constructed, based on the analysis of which they were assigned to GOX. Four sequences were selected from the genome databases of MSU and the FRC Biotechnology RAS after expert evaluation: one each from the bacteria Natronosporangium hydrolyticum ACPA39 [24] and Natroglycomyces albus ACPA22 [25] (this enzyme ended up being a glycine oxidase) and two from the archaea Natrarchaeobius halalkaliphilus AArcht4 [26]. In addition, sequences from two pathogens, Mycobacterium tuberculosis (MycDAAO) and Pseudomonas aeruginosa (PaeGOX), also found by our homology search, are presented in the alignment. In the NCBI database, the P. aeruginosa protein is annotated as DAAO.

Multiple alignment of the selected sequences was performed using Clustal X 1.83 software (Fig. 1). This program is used because it itself builds a hierarchy in the homology of the given sequences. The results of this alignment gave quite expected results. As shown in Fig. 1, depending on the source, the enzymes are clearly divided into two groups: bacterial DAAOs are at the top, followed by yeast and fungal enzymes, immediately followed by DAAO from archaea, and then by glycine oxidases. The second interesting point is that the widely used and well-studied TvaDAAO has the highest homology with bacterial DAAOs, while the second, even more thoroughly studied RtoDAAO, is at the very end of the list before archaea.

**Fig. 2:**
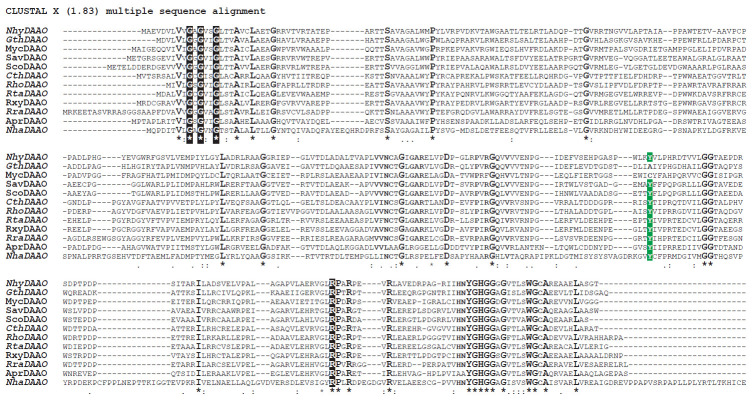
Alignment of amino acid sequences of D-amino acid oxidases from bacteria and archaea after screening out glycine oxidase sequences. See *Table 1* for the name correspondence. New DAAO sequences from bacteria analysed in this work are shown in bold italics. The Tyr residue that was previously considered conserved for binding D-amino acids in the active site is shown with letters on a green background in the alignment

As already mentioned, when searching for the genes of target enzymes in new sources, an approach based on the homology of proteins that perform the same function (e.g., catalyze the same reaction) is used. In some cases, such enzymes have very high homology in the substrate-binding and catalytic domains, and solving such a problem is not very difficult. A good example is NAD(P)+-dependent formate dehydrogenase (FDH), which consists of two identical subunits and does not have cofactors in the active center. The degree of homology between FDHs even from evolutionarily distant sources (e.g., bacteria and higher plants) is at least 55%, and a large number of sufficiently extended (up to 10–15 amino acid residues) conserved sequences in all parts of the active center are observed in multiple alignments [27, 28, 29]. In DAAO, the level of homology does not exceed 30%, which is much lower. In this case, information on the conserved and catalytically important amino acid residues could help to annotate the gene. However, in the case of DAAO, this approach is of little use. A characteristic feature of the catalytic mechanism of FAD-containing enzymes is the transfer of the hydride ion from the substrate to the isoalkoxazine ring of the cofactor proceeds without significant participation of the amino acid residues of the enzyme, whose main role is to form the proper conformation of the active centre necessary for catalysis and the participation of a number of residues in the binding of FAD and D-amino acids. In the case of a cofactor, the fingerprint sequence GxGxxG must be present at the N-terminus of the enzyme [30]. The presence of arginine and tyrosine residues (R285 and Y223 in RtoDAAO and R302 and Y243 in TvaDAAO) in the active site was considered mandatory for binding the carboxyl group of the D-amino acid. Similar residues are also present in mammalian enzymes [1, 2]. However, the expansion of the set of compared sequences indicates that only the fingerprint sequence in the FAD-binding domain and the arginine residue participating in binding the carboxyl group remain conserved. Note that the mobility of this Arg residue is strongly restricted by the neighboring conserved proline residue (ArgPro pair, Fig. 1, fourth row of alignment). The tyrosine residue (Fig. 1, third row of alignment, middle) is not conserved – two of the six OpaDAAOs as well as two bacterial enzymes, MycDAAO and GthDAAO, have different residues, Met, Phe, Ala, and Cys, in this position. In addition, these two features cannot be used to annotate the enzyme as a DAAO, since the same pair (the fingerprint sequence and the pair of conserved ArgPro residues) is present in all glycine oxidases. When only bacterial DAAO sequences are aligned (Fig. 2), the situation is more optimistic. As follows from Fig. 2, the region of the conserved ArgPro pair for all DAAOss in bacterial enzymes expands to the GxRPxR sequence, and a new conserved sequence YGHGGxG also appears. However, some glycine oxidases also have such sequences (not shown in Fig. 2).

One of the sequences found belongs to DAAO from the archaea N. halalkaliphilus AArcht4 (NhaDAAO). Figure 2 clearly shows that the amino acid sequence of this enzyme is longer than that of the bacterial DAAOs. The alignment clearly shows three large inserts in the region of the FAD- and substrate-binding domains, as well as at the C-terminus. However, NhaDAAO contains the same set of conserved residues as bacterial DAAOs. In the second analysed enzyme from N. halalkaliphilus AArcht4, glycine oxidase NhaGOX, the positions of insertions and deletions coincided very well with similar positions in GOX from P. aeruginosa (Fig. 1) and other glycine oxidases (not shown).

The results of the comparison of the amino acid sequences allow us to make some assumptions about the type of substrate specificity. If the size of the substrates differs significantly, one can easily notice differences in the length of the regions that form the substrate-binding domain, already at the amino acid sequence alignment stage. For example, TvaDAAO, RemDAAO, and FsoDAAO have longer sequences in the region of residues 100–108 (Fig. 1, left side of the second row of alignment). This is because TvaDAAO and FsoDAAO are able to oxidize bulk cephalosporin C, while other DAAOs that have deletions in this part of the alignment do not oxidize cephalosporin C. For example, CboDAAO is specific to small amino acids, primarily D-Ala [17]. However, it should be noted that a homology search does not distinguish the classical DAAO with a wide spectrum of substrate specificity from the D-amino acid oxidase DASPO, which is specific only to D-Asp and D-Glu. For example, OpaDAAO1 (Table 1) is listed in the O. parapolymorpha DL-1 genome annotation as D-aspartate oxidase (DASPO), although our experimental data indicate that this is absolutely not the case. Our results showed that the enzyme has a wide range of substrate specificity and is identical to RtoDAAO and TvaDAAO in pH profiles of activity and stability. A similar situation is observed in the annotation of the Pichia pastoris genome. PpaDAAO1, annotated as a D-amino acid oxidase, is actually DASPO, while PpaDAAO_2_, annotated as a “hypothetical protein with low homology to D-amino acid oxidase,” is exactly DAAO [14].

**Table 1:** D-amino acid oxidases and glycine oxidases and their sources^*^

No.	Short name	Source	Protein code in database
NCBI (GeneBank, UniProt)			
Bacteria			
1	GthDAAO	Gandjariella thermophila	WP_137812914.1
2	CthDAAO	Chloracidobacterium thermophilum B	WP_014099936.1
3	RtaDAAO	Rubrobacter taiwanensis	WP_132692836.1
4	RraDAAO	Rubrobacter radiotolerans DSM 5868	WP_084263988.1
5	RbaDAAO	Rhodothermaceae bacterium RA	ARA94025.1
6	RxyDAAO	Rubrobacter xylanophilus	BAP18969.1
7	MycDAAO	Mycobacterium tuberculosis	WP_003899072
8	SavDAAO	Streptomyces avermitilis MA-4680	BAC69383
9	ScoDAAO	Streptomyces coelicolor A3(2)	CAB40690
10	AprDAAO	Arthrobacter protophormiae	AY306197
11	PaeGOX	Pseudomonas aeruginosa	AAP81270
Fungi and yeasts			
12	TvaDAAO	Trigonopsis variabilis	AY514426
13	NcrDAAO	Neurospora crassa	EAA33029
14	FsoDAAO	Fusarium solani	BAA00692
15	RemDAAO	Rasamsonia emersonii	BBH51408
16	CboDAAO	Candida boidinii	BAB12222
17	PpaDAAO	Pichia pastoris CBS7435	SCV12162
18	SpoDAAO	Schizosaccharomyces pombe	NP_001342883
19	RtoDAAO	Rhodosporidium toruloides (Rhodotorula gracilis)	U60066
20	OpaDAAO1	Ogataea parapolymorpha DL-1	XP_013932717
21	OpaDAAO2	Ogataea parapolymorpha DL-1	XP_013937260
22	OpaDAAO3	Ogataea parapolymorpha DL-1	XP_013934816
23	OpaDAAO4	Ogataea parapolymorpha DL-1	XP_013937224
24	OpaDAAO5	Ogataea parapolymorpha DL-1	XP_013937169
25	OpaDASPO	Ogataea parapolymorpha DL-1	XP_013932178
The genome database of the Lomonosov Moscow University and the Federal Research Center of Biotechnology			
Bacteria			
26	NhyDAAO	Natronosporangium hydrolyticum ACPA39	lcl|CP070499.1_prot_QSB16697.1_2115
27	NalGOX	Natroglycomyces albus ACPA22	lcl|CP070496.1_prot_QSB06127.1_824
Archaea			
28	NhaDAAO	Natrarchaeobius halalkaliphilus AArcht4	2642575300
29	NhaGOX	Natrarchaeobius halalkaliphilus AArcht4	2642575587

^*^The new sequences of DAAOs from extremophilic microorganisms analysed in this study are shown in bold italics.

**Construction of model 3D structures and their comparative analysis with known DAAO structures **As already noted in the Introduction, the goal of searching for and cloning new genes is not just to obtain a recombinant enzyme but to create a biocatalyst with desired properties using the closest enzyme to the target as the initial one. In the case of DAAO, it is simply impossible to draw a conclusion about the properties (primarily about the substrate specificity and the optimal pH profile of activity) based on the alignment. In this regard, the use of additional methods is required. To solve this problem, we proposed an approach based on 3D structure modelling. In the first stage, model structures of new enzymes are compared with experimental and model structures of known D-amino acid oxidases and glycine oxidases.

Many examples have been published where enzymes with low homology have very close spatial structures. A good example is the supersecondary structure called the Rossman fold, which is universal for binding the adenine part of various cofactors and coenzymes, such as NAD(P)+, FAD, ATP, SAM, etc. [31]. Using this approach to DAAO until recently was impossible due to the lack of a representative set of structures. Experimental structures were solved for only four enzymes: yeast RtoDAAO and RemDAAO and enzymes from pig kidney (pkDAAO) and humans (hDAAO). A model structure of TvaDAAO was constructed [32]. However, this enzyme is very similar to RemDAAO in both its primary (Fig. 1) and tertiary (Table 2) structures. In addition, the previously used modelling methods gave good results only at high sequence homology between the studied enzyme and the enzyme whose structure is used as a template for constructing a 3D model structure. High accuracy was achieved with a homology of at least 50–60%, which is not observed in the case of DAAO. The situation changed dramatically when a new algorithm for model structure construction, AlphaFold, was proposed in 2021 [33]. In 2022, the prediction accuracy was significantly improved [19]. The use of AlphaFold2 makes it possible to obtain reliable information on the structure of both new enzymes and already described DAAOs. Such model structures were constructed in our work. The structures of 18 DAAOs (including eight new ones) have been modelled. Table 2 shows the results of a pairwise comparison of model and experimental structures of D-amino acid oxidases and glycine oxidases. In this case, the set of analysed DAAO structures was extended with two experimental mammalian DAAO structures, from pig and human kidney, as well as with the structures of two glycine oxidases. In addition, our own preliminary data from the X-ray diffraction analysis of TvaDAAO and OpaDAAO1 were also used for comparison. Such an extended set allows more accurate comparison and increases the reliability of assigning new proteins to DAAO or GOX. For convenience, the comparison results shown in Table 2 are highlighted in color. The green background shows the results comparing structures with RMSD up to 1 Å, light green with RMSD from 1 to 2 Å, light orange with RMSD from 2 to 6 Å, and orange with RMSD above 6 Å. Several important and interesting results of the analysis of the data in Table 2 can be noted.

**Table 2:**
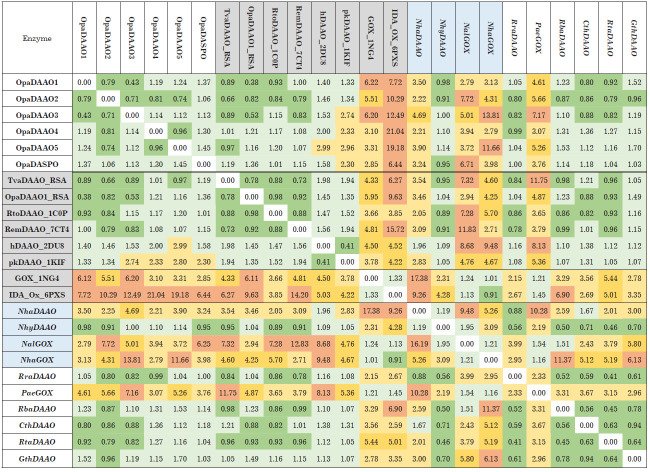
The standard deviation between the structures of D-amino acid oxidases and glycine oxidases^*^. ^*^New DAAO sequences analysed in this study are shown in bold italics.

(1) The latest modification of the AlphaFold algorithm in the 2022 version [19] truly allows one to obtain model structures with very high accuracy. This is clearly seen when comparing the model and experimental structures of OpaDAAO1. The RMSD between these structures is only 0.38 Å. The RMSD between the model and experimental TvaDAAO structures is slightly larger, 0.56 Å (not shown in Table 2), but it should be taken into account that these enzymes have different oligomeric structures (OpaDAAO1 is a monomer, TvaDAAO is a dimer). The high accuracy of OpaDAAO1 structure prediction leads to the fact that a pairwise comparison of the model and experimental structures of OpaDAAO1 with the model and experimental structures of other enzymes gives almost identical RMSD values (Table 2, lines 1 and 8).

(2) There is a clear correlation between the function and overall structure of D-amino acid oxidases and glycine oxidases. The value of RMSD deviation between DAAO structures does not exceed 2 Å, while when comparing DAAO and GOX structures, the RMSD value is 3 Å or more (up to 15–18 Å). The results with NhaDAAO from archaea slightly fall out of the general picture – the deviation of the model structure from the structures of other DAAOs is 2.0–3.5 Å (in the case of OpaDAAO3, the deviation reaches even 4.69 Å). At the same time, the difference in the structure of NhaDAAO with glycine oxidases is much greater, from 9 to 17 Å. We should also note that this enzyme has a general structure close to that of human DAAO (RMSD is only 1.96 Å). Such results indicate that to correctly confirm that this enzyme is a DAAO, as broad a set of structures of known D-amino acid oxidases as possible should be used. Nevertheless, although the results of a general comparison of the structure of NhaDAAO with the structures of other DAAOs are generally slightly outside the 2 Å boundary value, the homology analysis and comparison of the general structure allowed us to classify this enzyme as a D-amino acid oxidase. The results of comparison of the structures of active centers fully confirm this conclusion.

**Comparative Analysis of the Structures of DAAO Active Centers**

**Fig. 3:**
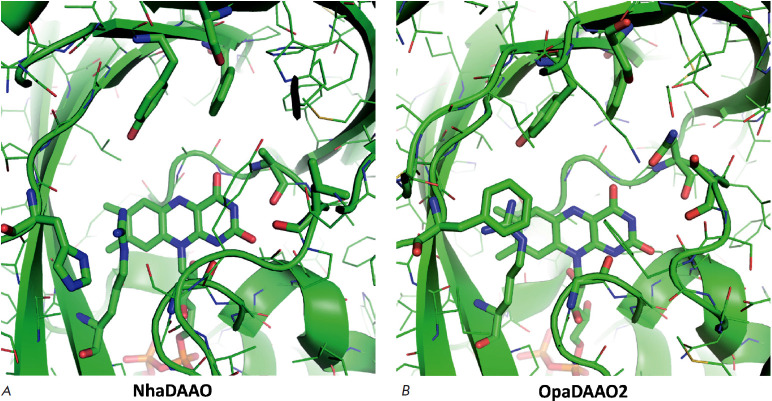
The model structures of the active sites of *NhaDAAO *from the archaea *N. halalkaliphilus *AArcht4 (*A*) and OpaDAAO_2_ from the methylotrophic yeast *O. parapolymorpha *DL-1 (*B*)

In the next step, we compared the structures of the active centers of the new DAAOs with the known D-amino acid oxidases. The coincidence of the structure of the new protein with the structure of the active center of known enzymes clearly shows that the protein of interest belongs to certain enzyme families. The structure of the FAD-binding domain should be very similar in all DAAOs, but due to different specificities, the structure of substrate-binding domains should differ quite significantly both in volume and in the type of residues involved in the binding of a particular D-amino acid. Therefore, the coincidence of the structures of the substrate-binding domains of the active center allows one to unambiguously prove that the new enzyme belongs to the DAAO family and to draw a fairly reliable conclusion about the possible spectrum of substrate specificity. In this case, a comparison with the structures of DAAO active centers from the yeast O. parapolymorpha DL-1 is particularly useful since these enzymes differ greatly from each other both in the profile of substrate specificity and in the pH dependences of activity and stability. The effectiveness of such a comparison is clearly seen by the example of NhaDAAO from archaea. As noted above, this enzyme differs rather markedly from other DAAOs both in its amino acid sequence length and in its overall structure. However, the results of a comparison of the structures of the active sites indicate that NhaDAAO and OpaDAAO_2_ have almost identical active centers (Fig. 3). Alignment of the overall structures with the FAD cofactor reveals that in addition to the conserved Arg residue in the substrate-binding domain (see above), there are Tyr and Phe residues involved in substrate binding, and the locations of these residues in the active centers of NhaDAAO and OpaDAAO_2_ are almost identical. Moreover, the results of modelling the structure of the active center of OpaDAAO_2_ itself are in complete agreement with the experimental data, according to which the best substrates are D-amino acids with hydrophobic side groups – D-Phe (the highest activity), D-Tyr and D-Leu. Therefore, it is logical to assume that NhaDAAO should have the same spectrum of substrate specificity. High specificity to D-Leu and D-Phe was also predicted by comparison with the active center of OpaDAAO3 and the enzyme from N. hydrolyticum ACPA39 (NhyDAAO) (not shown). At present, the gene of this enzyme has been cloned in our laboratory, and its expression in E. coli cells is in progress. Preliminary experiments confirmed that D-Leu and D-Phe are the best substrates for NhyDAAO (a detailed description of the preparation and study of the properties of recombinant NhyDAAO will be presented in a separate publication).

**Fig. 4:**
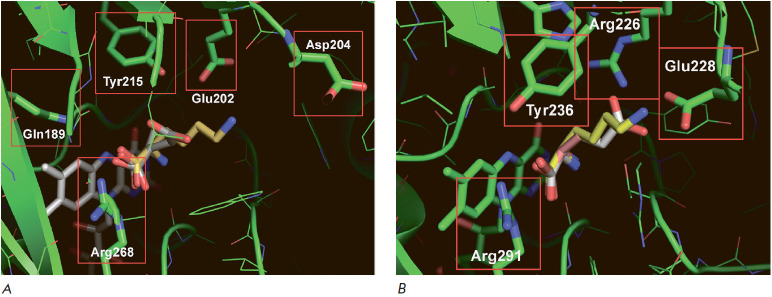
Docking of D-Ala, D-Asp and D-Lys to the DAAO active site from *G. thermophila *(*A*) and D-Ala, D-Glu and D-Lys to the DAAO active site from *R. radiotolerans *DSM 5868 (*B*)

A comparison of DAAO structures showed that the active centers of enzymes from G. thermophila (GthDAAO) and from R. radiotolerans DSM 5868 (RraoDAAO) are quite unique. In GthDAAO, the carboxyl groups of the side chain of residues Glu202 and Asp204 must participate in substrate binding (Fig. 4A). This suggests that this enzyme can be specific to D-Lys and D-Arg, but docking of various D-amino acids indicates that both carboxyl groups of residues Glu202 and Asp204 are located quite far away (more than 3 Å) from the substrate molecule. A more interesting situation is observed in the case of RraDAAO (Fig. 4B). The positively charged residue Arg226 and the negatively charged residue Glu228 can participate in the binding of the substrate side groups. Docking to the active center of various D-amino acids suggests that RraDAAO should be specific to positively charged D-Lys and potentially active with D-Glu. Cloning of the gene for this enzyme is of interest because D-Lys is a poor substrate for all DAAOs described.

## CONCLUSIONS

The results of our experiments allow us to draw several conclusions.

(1) The introduction of the second stage – structural analysis – in the identification of genes for new D-amino acid oxidases, after a search in genomes by homology, is a highly effective and necessary procedure. At this stage, it is possible not only to unambiguously confirm that the new enzyme belongs to DAAO but also to predict the possible spectrum of its substrate specificity. The reliability of such prediction of high activity with D-Leu and D-Phe for new DAAO from the bacterium from N. hydrolyticum ACPA39 was confirmed experimentally.

(2) The amino acid sequences of D-amino acid oxidases from bacteria have low homology (no more than 30%). Analysis of the bacterial DAAO sequences revealed new characteristic conserved elements that can be used for identification of these enzymes during their search in bacterial genomes. The presence of new conserved regions was also shown in the DAAO sequence of N. halalkaliphilus AArcht4 (NhaDAAO) archaea.

(3) The D-amino acid oxidase gene was found in the archaean genome for the first time. Compared to bacterial DAAOs, the NhaDAAO enzyme from archaea has a longer amino acid sequence and less similar overall three-dimensional structure, but the results of structural analysis clearly showed that the active center of NhaDAAO is almost identical to the active center of OpaDAAO_2_ from the methylotrophic yeast O. parapolymorpha DL-1. Additionally, a glycine oxidase was identified in the genome of N. halalkaliphilus AArcht4, which is the closest in homology to GOX from the pathogen P. aeruginosa.

(4) D-amino acid oxidases play an important role in the functioning of microorganisms and mammals. That is why the search for human hDAAO inhibitors is one of the most active and topical areas of research on this enzyme [34]. Reliable identification of the D-amino acid oxidase gene (MycDAAO) in the genome of the tuberculosis causative pathogen allows us to consider this enzyme as a target for the development of a new type of drug against tuberculosis. Due to the rare occurrence of DAAO in bacteria and due to the significant differences of this enzyme from other DAAOs (primarily from hDAAO), efficient inhibitors that bind specifically to MycDAAO can be used as anti-tuberculosis drugs.

## Abbreviations

DAAO: D-amino acid oxidase
GOX: glycine oxidase
